# Supplementary material for: Selective hydrogenation via precise hydrogen bond interactions on catalytic scaffolds
Source: Nat Commun. 2023 Jan 26;14:429. doi: 10.1038/s41467-023-36015-z (PMC9879947; doi:10.1038/s41467-023-36015-z)
Supplement: Supplementary file 1 — Supplementary Information [file 41467_2023_36015_MOESM1_ESM.pdf]

Supplementary Information

Selective hydrogenation via precise hydrogen bond interactions on catalytic scaffolds

Song Shi[1,2§], Piaoping Yang[1§], Chaochao Dun[3], Weiqing Zheng[1], Jeffrey J. Urban[3], Dionisios G. Vlachos[1*]

[1] Department of Chemical and Biomolecular Engineering and Catalysis Center for Energy Innovation (CCEI), University of Delaware, Newark, DE 19716, USA.

[2] State Key Laboratory of Catalysis, Dalian Institute of Chemical Physics, Chinese Academy of Sciences, Dalian 116023, People's Republic of China.

[3] The Molecular Foundry, Lawrence Berkeley National Laboratory Berkeley, CA 94720(USA)

[§] These authors contributed equally.

✉ email: vlachos@udel.edu

Table of contents

Figure S1. Wettability difference of support with different functional groups. 3
Figure S2. Pore structure comparison of different catalytic materials.. 4
Figure S3. Ir particle size. 5
Figure S4. Crystallinity of supports.. 6
Figure S5. Thermal stability of the two catalysts. 7
Figure S6. Kinetics and conditions for optimizing the reaction. 8
Figure S7. Reaction of 2-methylfuran.. 9
Figure S8. Electronic state of Ir over different supports via XPS 10
Figure S9. Electronic state of Ir over different supports via CO adsorption 11
Figure S10. Microscopy characterization of Pd and Pt catalyst. 12
Figure S11. XPS spectra of the Pd and Pt catalysts, ... 13
Figure S12. Adsorption isotherm of furfural on different supports. 14
Figure S13. Adsorption of toluene on HCP-OH and HCP-CH_3 15
Figure S14. Interaction of furfural with different supports, using *in situ* Drifts IR adsorption 16
Figure S15. Interaction of furfural with HCP-CH_3 via *in situ* Drifts-IR TPD experiments. .. 17
Figure S16. Interaction of furfural with HCP-OH, using *in situ* Drifts-IR TPD experiments. 18
Figure S17. Interaction of furfural with different supports, using ATR-IR adsorption. 19
Figure S18. Hydrogen bond measurement between furfural and phenol. 20
Figure S19. Hydrogen bond measurement between 2-MF and phenol. 21
Figure S20. Computational models. 22
Figure S21. Optimized configurations of furfural adsorption 23
Figure S22. Optimized configurations of the interaction between furfural and monomers ... 24
Figure S23. Particle size. 27
Figure S24. Ir particles after reaction. 28
Figure S25. Catalyst reusability of Ir-HCP-OH. 28
Figure S26. Hot filter experiment of furfural hydrogen with Ir-HCP-OH. 29

Figure S27. Ketone adsorption difference over different zeolites. 30
Figure S28. Surface hydroxy difference of Si-Beta and Si-ZSM-5. 31
Figure S29. Reaction networks for the reactions depicted in figures 2a, 2c, and 2d. 32
Table S1. Textural properties of HCP, Ir-HCP, Si-Beta, and ZSM-5. 33
Table S2. Adsorption area of cyclopentanone, cyclohexanone, and cycloheptanone on Si-Beta and Si-ZSM-5. 33
Table S3. Adsorption energies (E_{ads}) of furfural over OH group and CH_3 group on the zigzag model and armchair model. 34
Table S4. Comparation of recent works on furfural hydrogenation modulation. 34
Supplementary Note 1 35

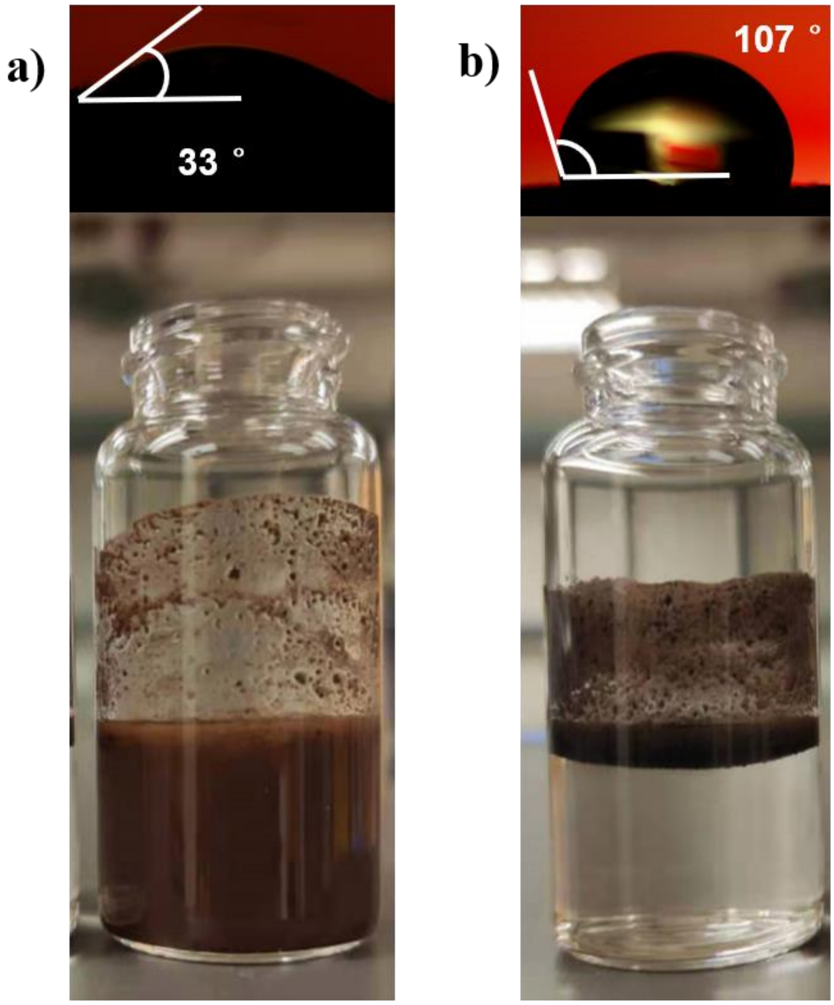

Figure S1. Wettability difference of support with different functional groups. HCP distribution difference on water and water contact angles. a) HCP-OH, b) HCP-CH_3.

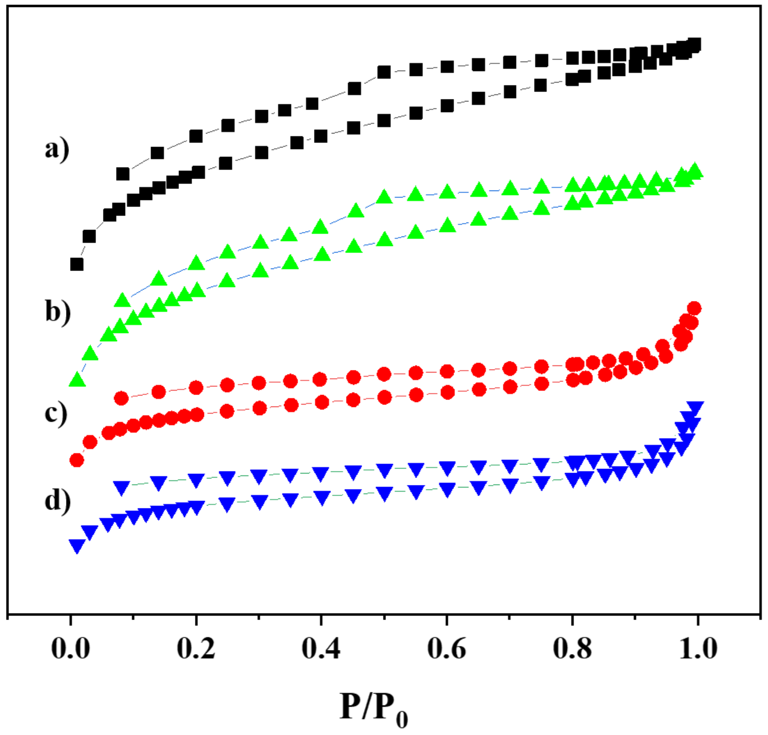

Figure S2. Pore structure comparison of different catalytic materials.

N_2 adsorption-desorption isotherms at 77 K. a) Ir-HCP-OH, b) HCP-OH, c) Ir-HCP-CH_3, d) HCP-CH_3.

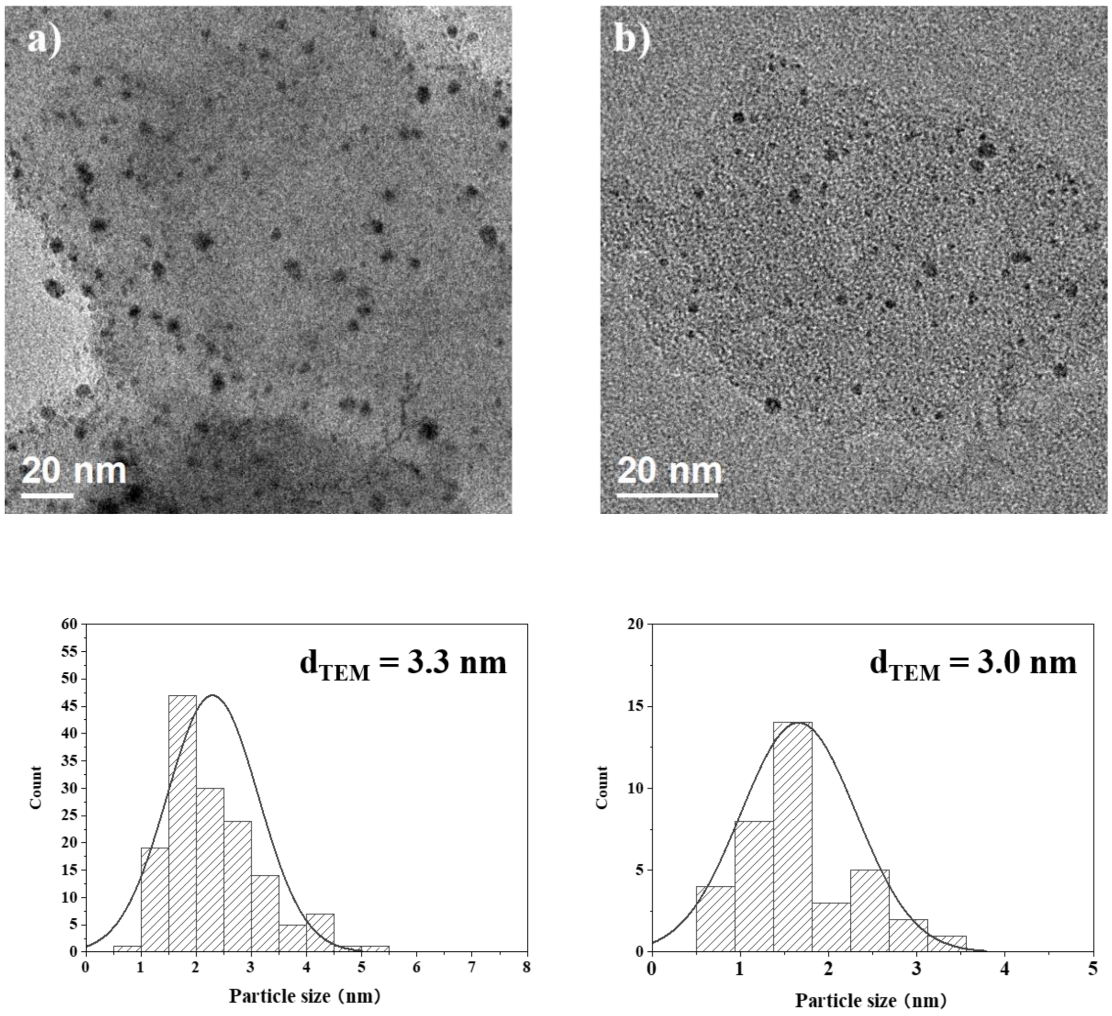

$$d_{TEM} = \sum n_i d_i^3 / \sum n_i d_i^2 \qquad \textbf{equation S1}$$

Figure S3. Ir particle size. TEM images and particle size distribution of a) Ir-HCP-OH and b) Ir-HCP-CH_3.

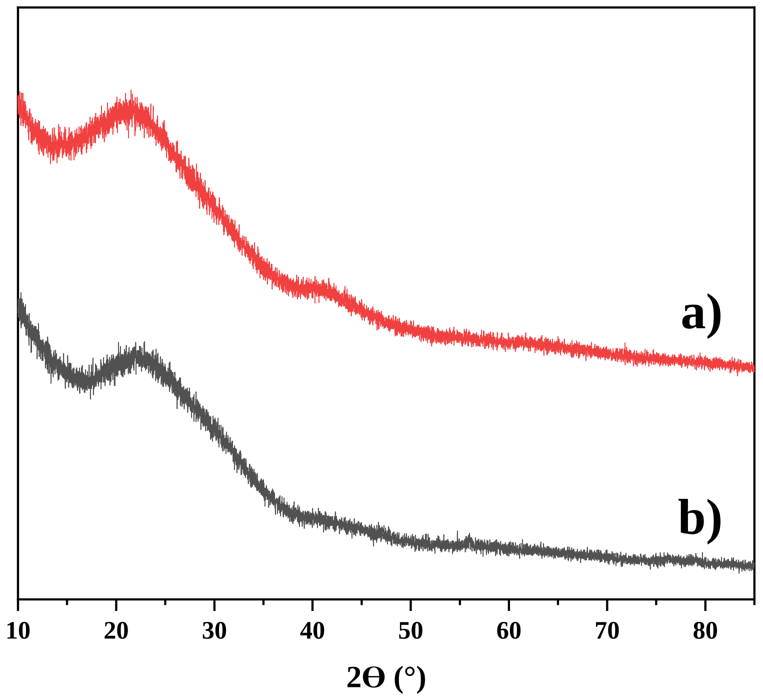

Figure S4. Crystallinity of supports. XRD patterns of a) HCP-CH_3 and b) HCP-OH.

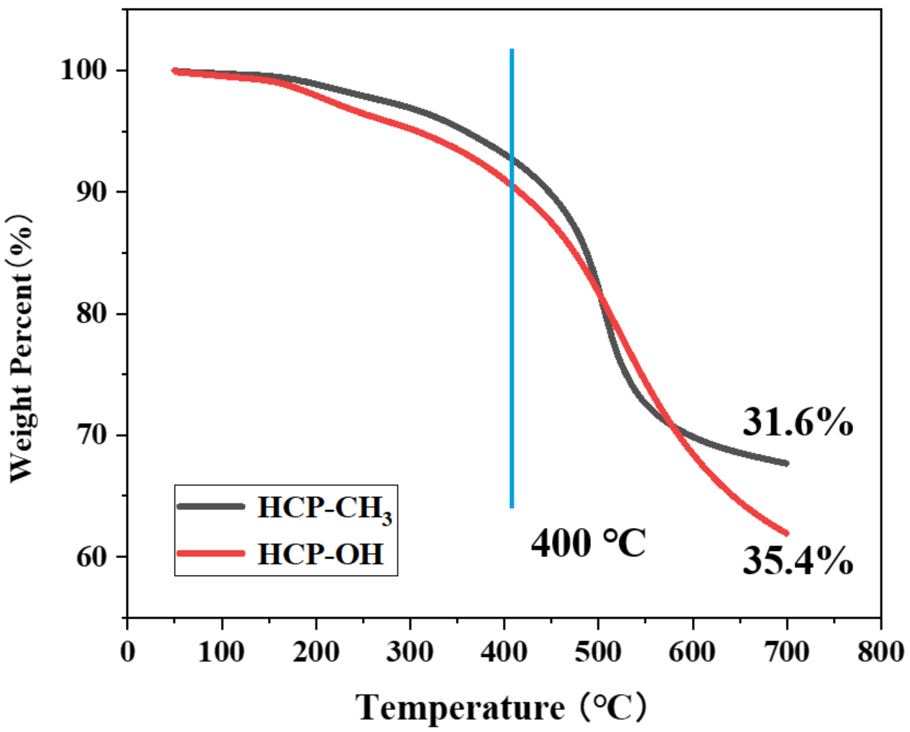

Figure S5. Thermal stability of the two catalysts. TGA curves of HCP-OH and HCP-CH_3.

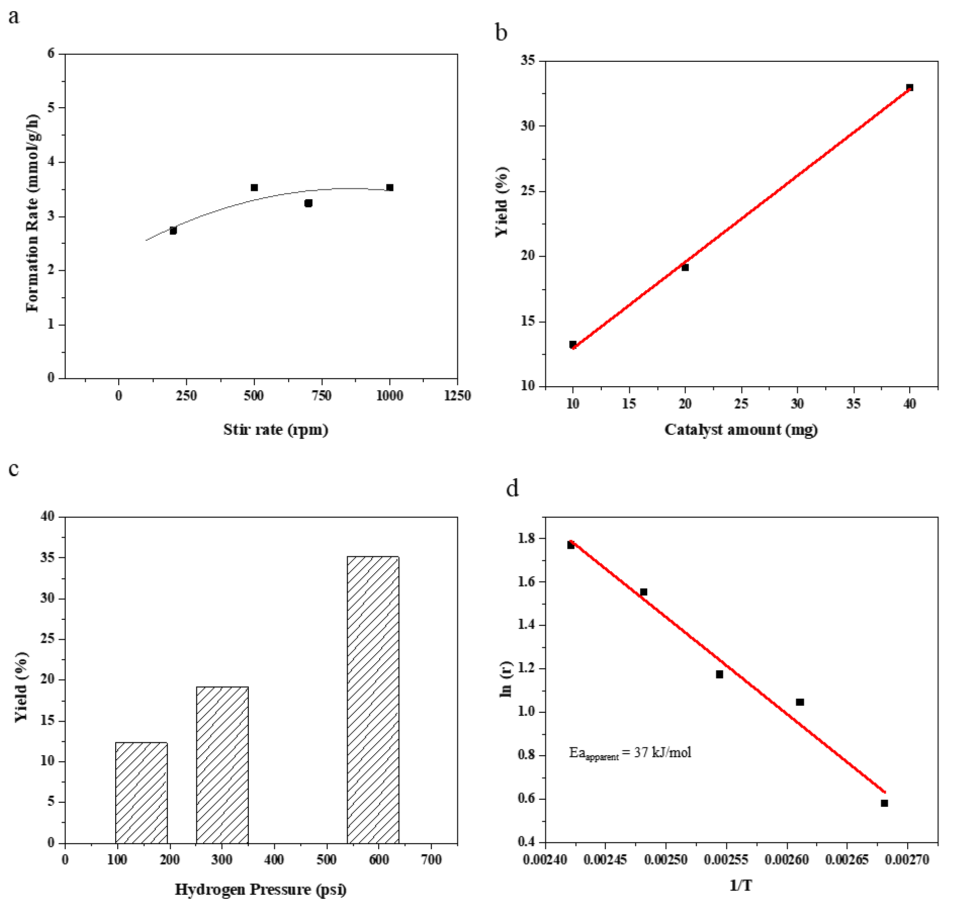

Figure S6. Kinetics and conditions for optimizing the reaction. a), Stir rate effect on rate with Ir-HCP-OH, b), catalyst amount effect with Ir-HCP-OH, c), hydrogen pressure effect with Ir-HCP-OH, d), Arrhenius plot of Ir-HCP-OH in furfural hydrogenation.

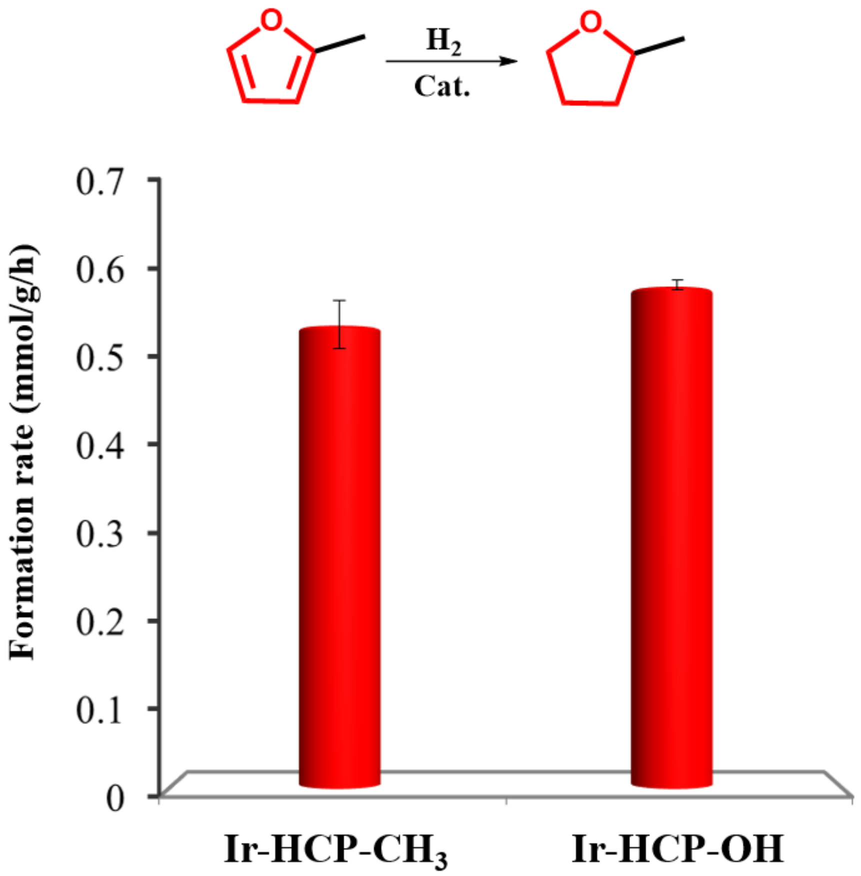

Figure S7. Reaction of 2-methylfuran. Hydrogenation rate over Ir-HCP-CH_3 and Ir-HCP-OH. Reaction conditions: substrate, 1 mmol; catalyst, 40 mg; hexane, 10 mL; H_2, 300 psi; 120 °C.

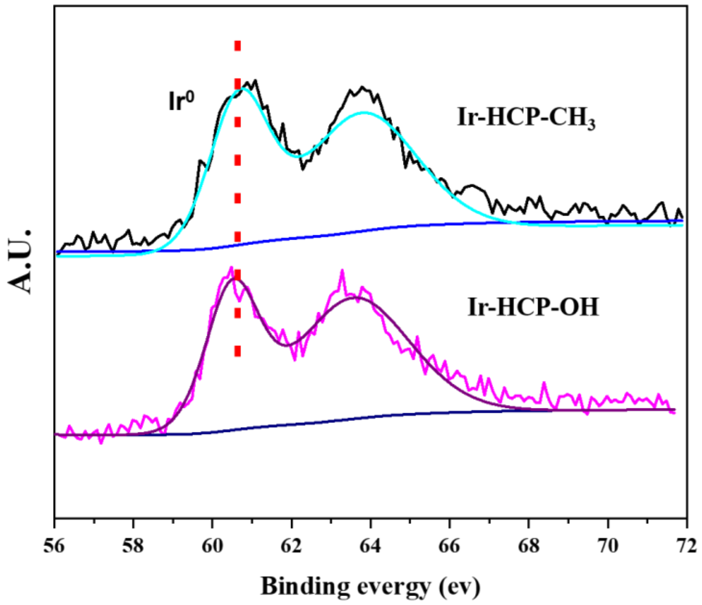

Figure S8. Electronic state of Ir over different supports via XPS. Ir *4f* of Ir-HCP-CH_3 and Ir-HCP-OH.

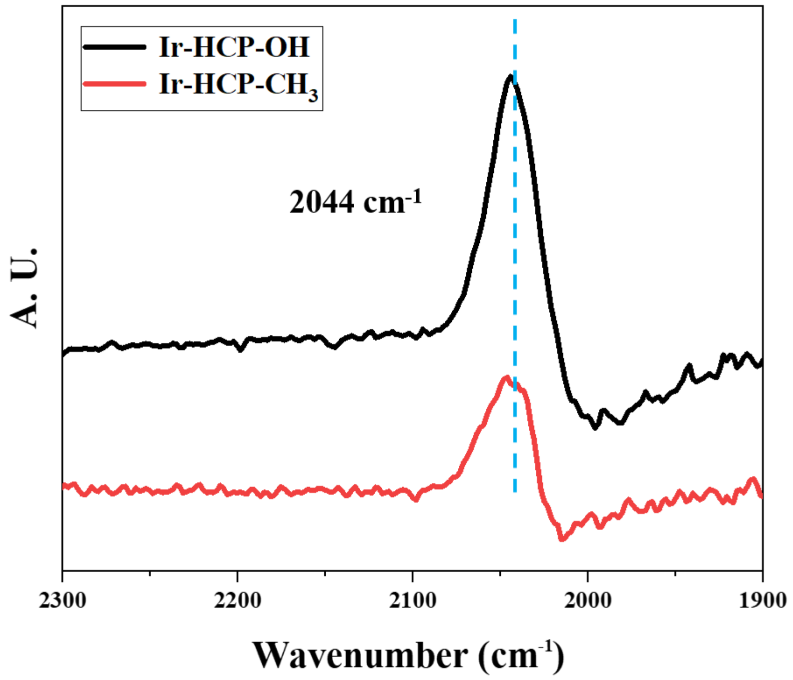

Figure S9. Electronic state of Ir over different supports via CO adsorption. *In situ* CO adsorption Drifts-IR peaks over Ir-HCP-CH_3 and Ir-HCP-OH.

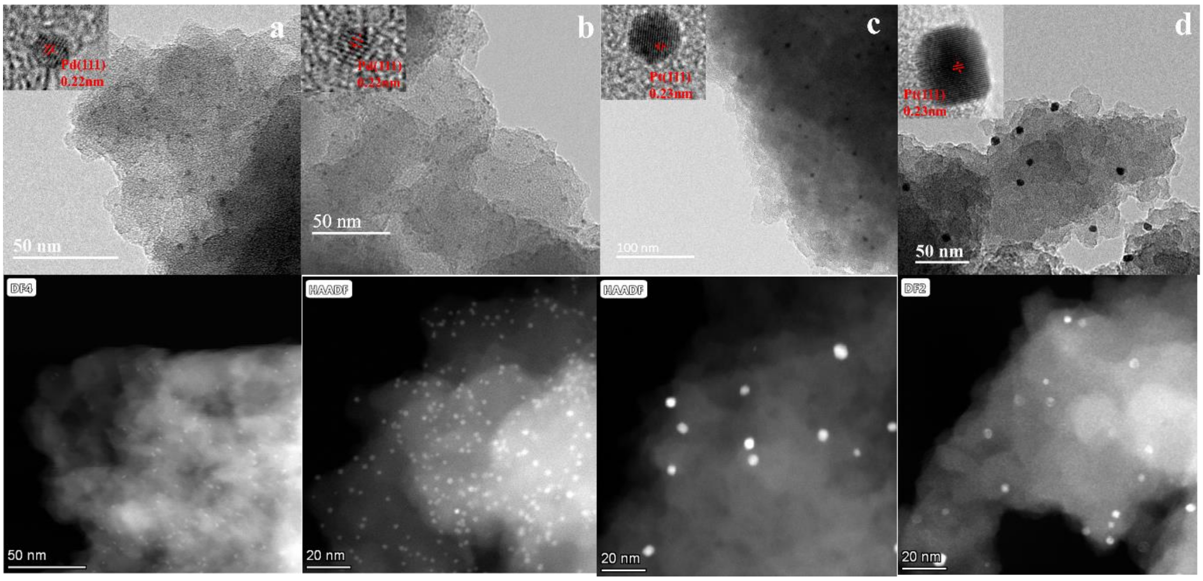

Figure S10. Microscopy characterization of Pd and Pt catalyst. TEM, HR-TEM and HAADF of a, Pd-HCP-CH_3, b, Pd-HCP-OH, c, Pt-HCP-CH_3, d, Pt-HCP-OH.

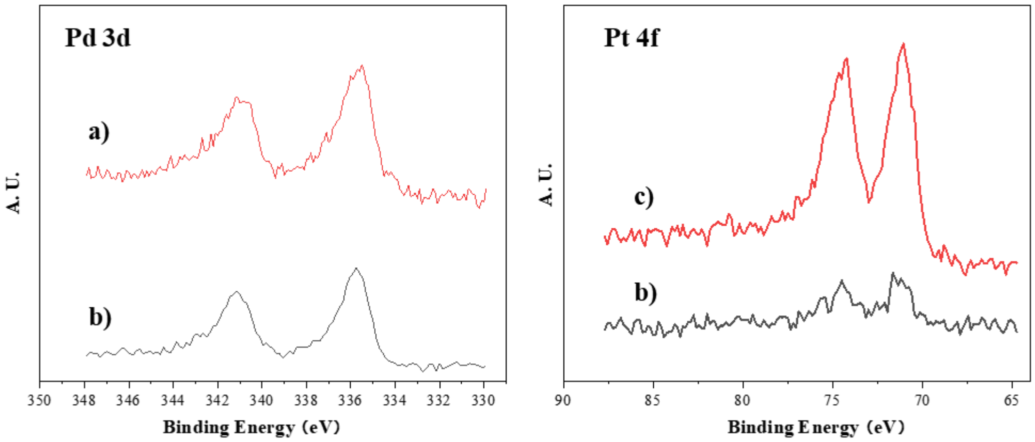

Figure S11. XPS spectra of the Pd and Pt catalysts, a) Pd-HCP-CH_3, b) Pd-HCP-OH, c) Pt-HCP-CH_3, and d) Pt-HCP-OH.

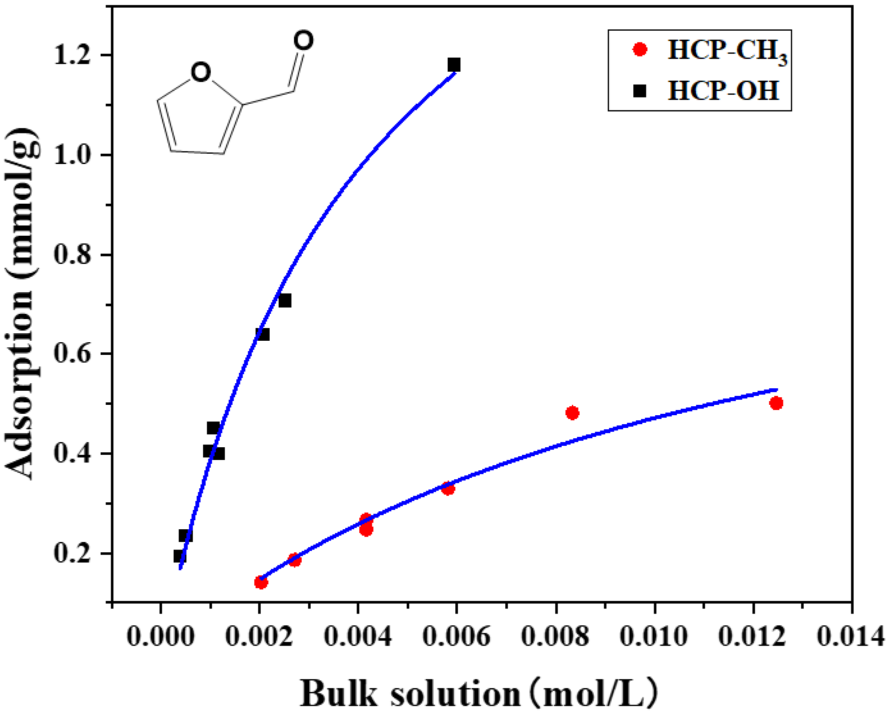

Furfural	Qmax (mmol/g)	K
HCP-OH	1.95 ±0.15	247±32
$HCP-CH_3$	1.05 ± 0.19	82±24

Figure S12. Adsorption isotherm of furfural on different supports. Results on HCP-OH and $HCP-CH_3$. Q_{max} and K are the saturated adsorption amount and affinity constant based on a Langmuir fit.

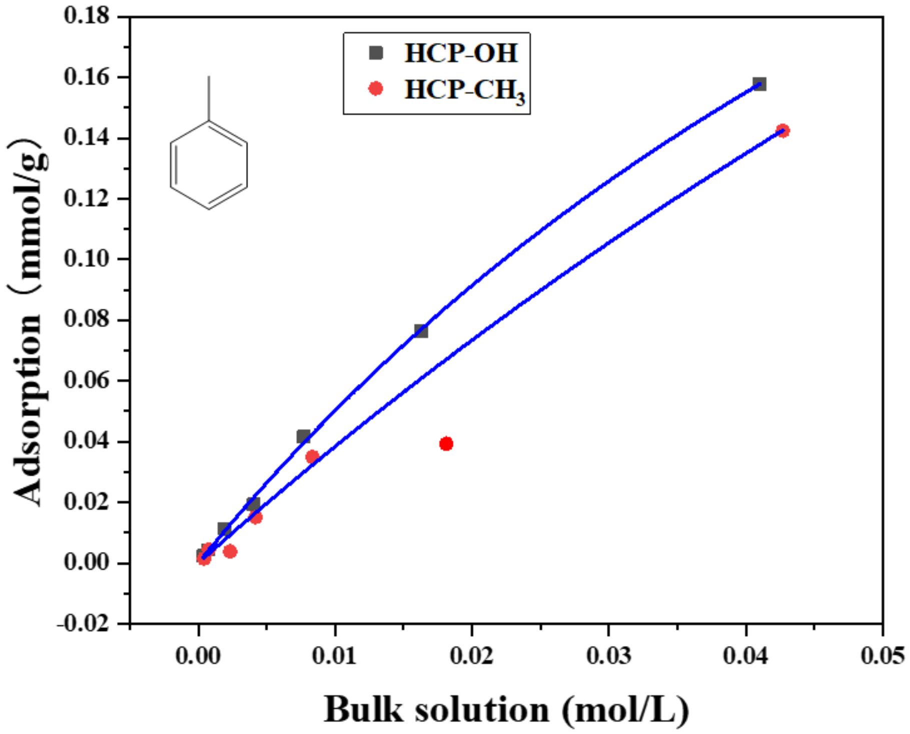

Toluene	Qmax (mmol/g)	K
HCP-OH	0.51±0.04	10.9±1
HCP-CH_3	0.83±0.44	4.9±3

Figure S13. Adsorption of toluene on HCP-OH and HCP-CH_3. Q_{max} and K are the saturated adsorption amount and affinity constant based on a Langmuir fit.

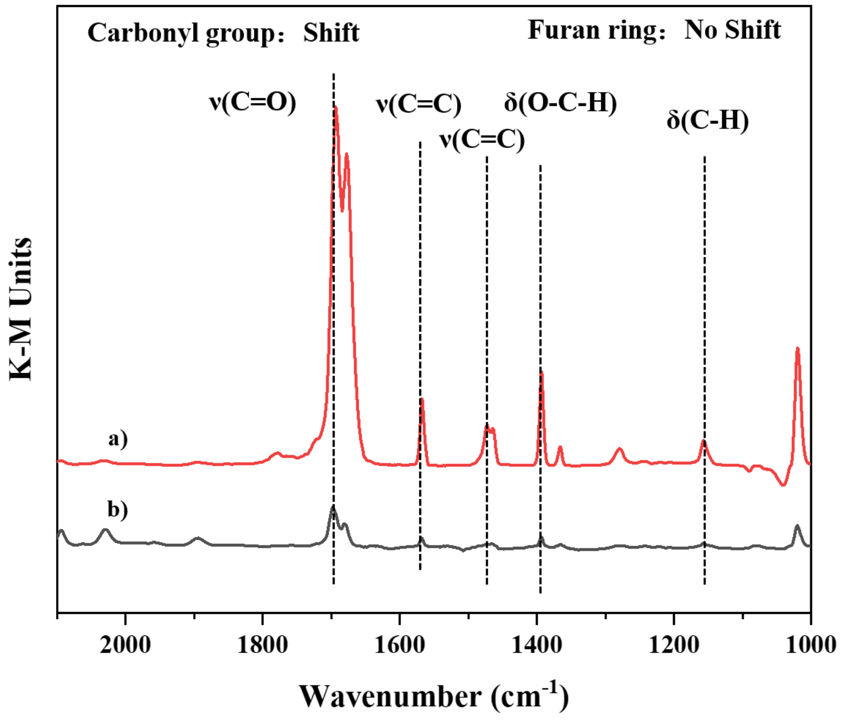

Figure S14. Interaction of furfural with different supports, using *in situ* Drifts IR adsorption. a) HCP-OH and b) HCP-CH_3 after the Kubelka-Munk (K-M) transformation.

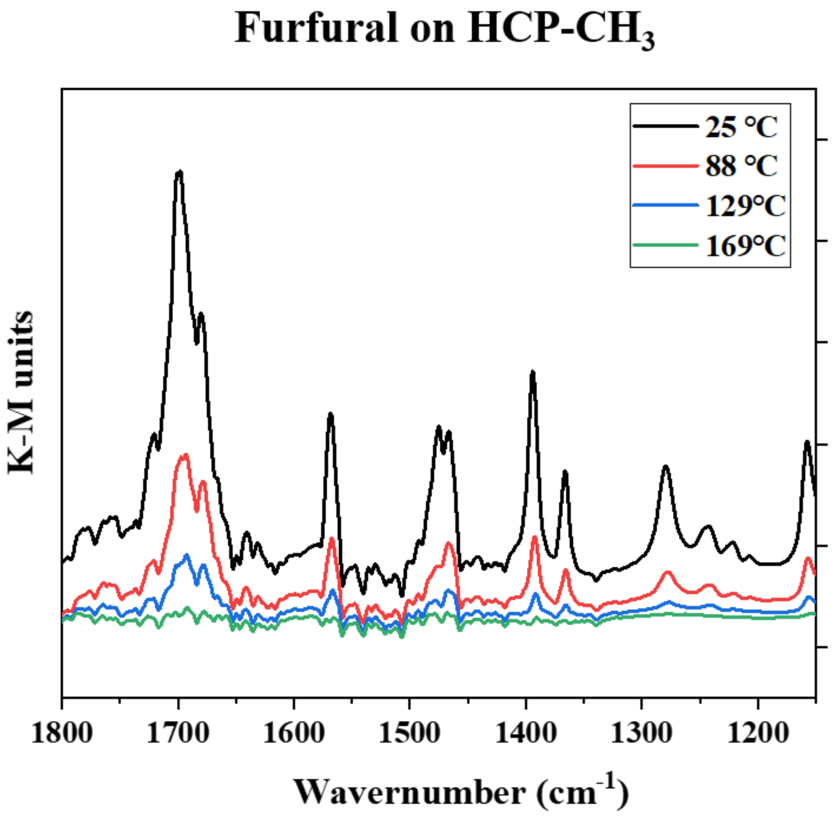

Figure S15. Interaction of furfural with HCP-CH_3 via *in situ* Drifts-IR TPD experiments. Results on HCP-CH_3 under different temperatures after the K-M transformation.

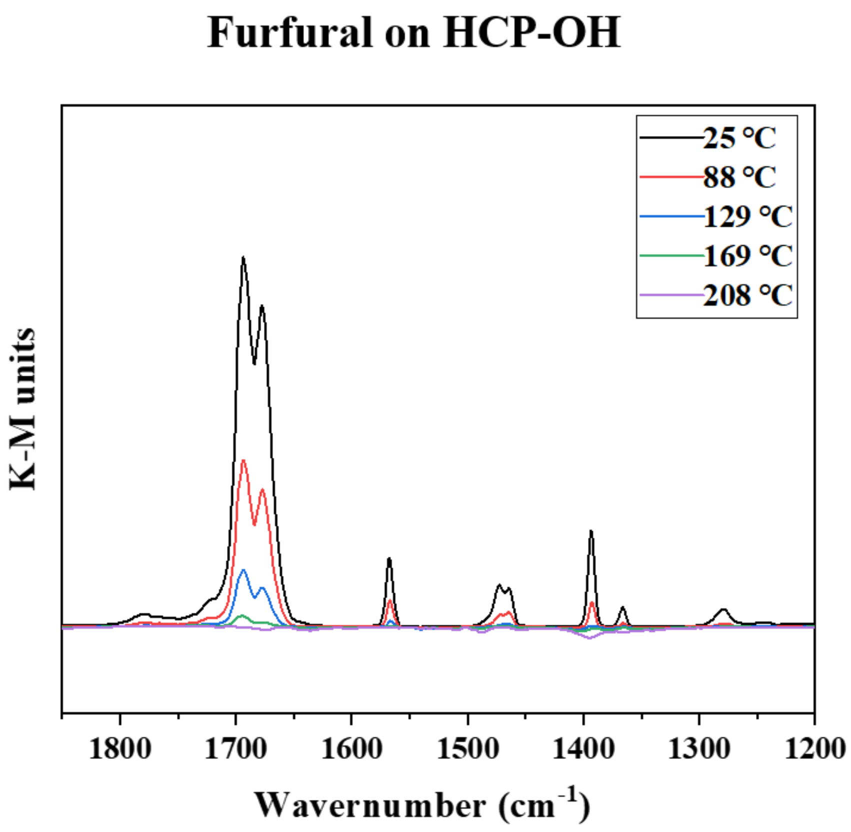

Figure S16. Interaction of furfural with HCP-OH, using *in situ* Drifts-IR TPD experiments. Drifts-IR of furfural on HCP-OH under different temperatures after the K-M transformation.

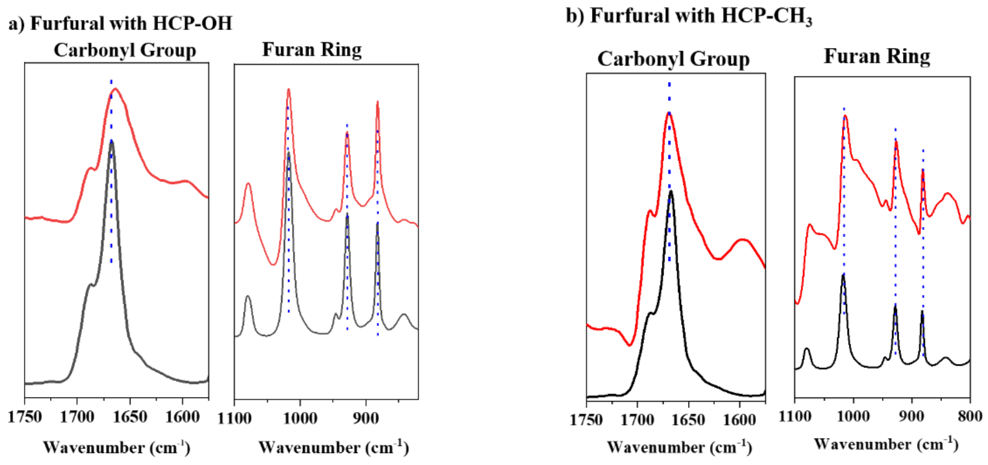

Figure S17. Interaction of furfural with different supports, using ATR-IR adsorption. a) HCP-OH and b) HCP-CH_3. Black line refers to the pure liquid furfural and the red line to furfural adsorbed on catalyst. Peaks at 1670 cm^{-1} are assigned to the carbonyl groups while those at 1018 cm^{-1}, 931 cm^{-1} and 886 cm^{-1} to the furan ring.

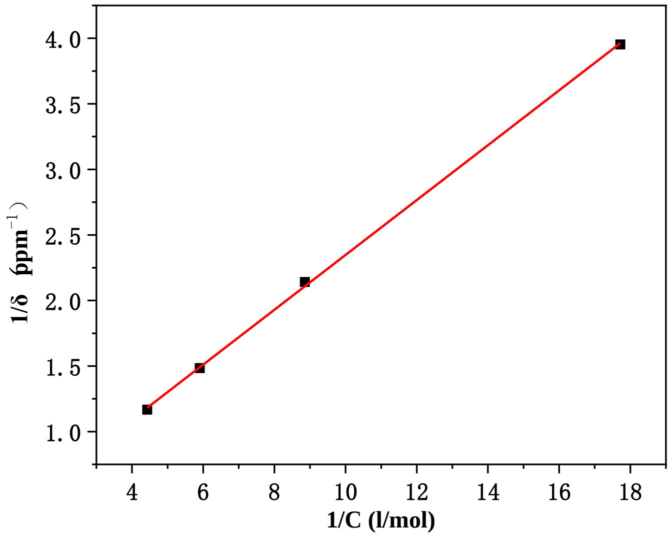

ratio	△δ（ppm）	δ（ppm）
0:1		4.5579
1:1	0.253	4.8109
2:1	0.4671	5.025
3:1	0.6739	5.2318
4:1	0.8566	5.4145
△G	-0.552 Kcal/mol	

$1/\Delta\delta = a \times 1/K \times 1/C + a$ **equation S2**

$\Delta G = -RT\ln(K)$ **equation S3**

Figure S18. Hydrogen bond measurement between furfural and phenol. Linear relationship between 1/Δδ and 1/C of furfural and phenol in $CDCl_3$, and chemical shift of active H in phenol with the addition of furfural.

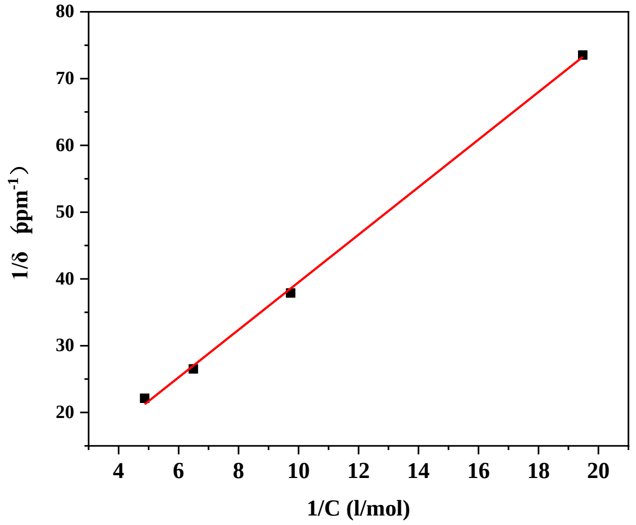

Ratio	△δ (ppm)	δ (ppm)
0:1		4.5579
1:1	0.0136	4.5715
2:1	0.0264	4.5843
3:1	0.0377	4.5956
4:1	0.0452	4.6031
△G	-0.213 Kcal/mol	

$1/\Delta\delta = a \times 1/K \times 1/C + a$ **equation S2**

$\Delta G = -RT\ln(K)$ **equation S3**

Figure S19. Hydrogen bond measurement between 2-MF and phenol.

Linear relationship between $1/\Delta\delta$ and $1/C$ of 2-MF and phenol in $CDCl_3$, and chemical shift of active H in phenol with the addition of 2-MF.

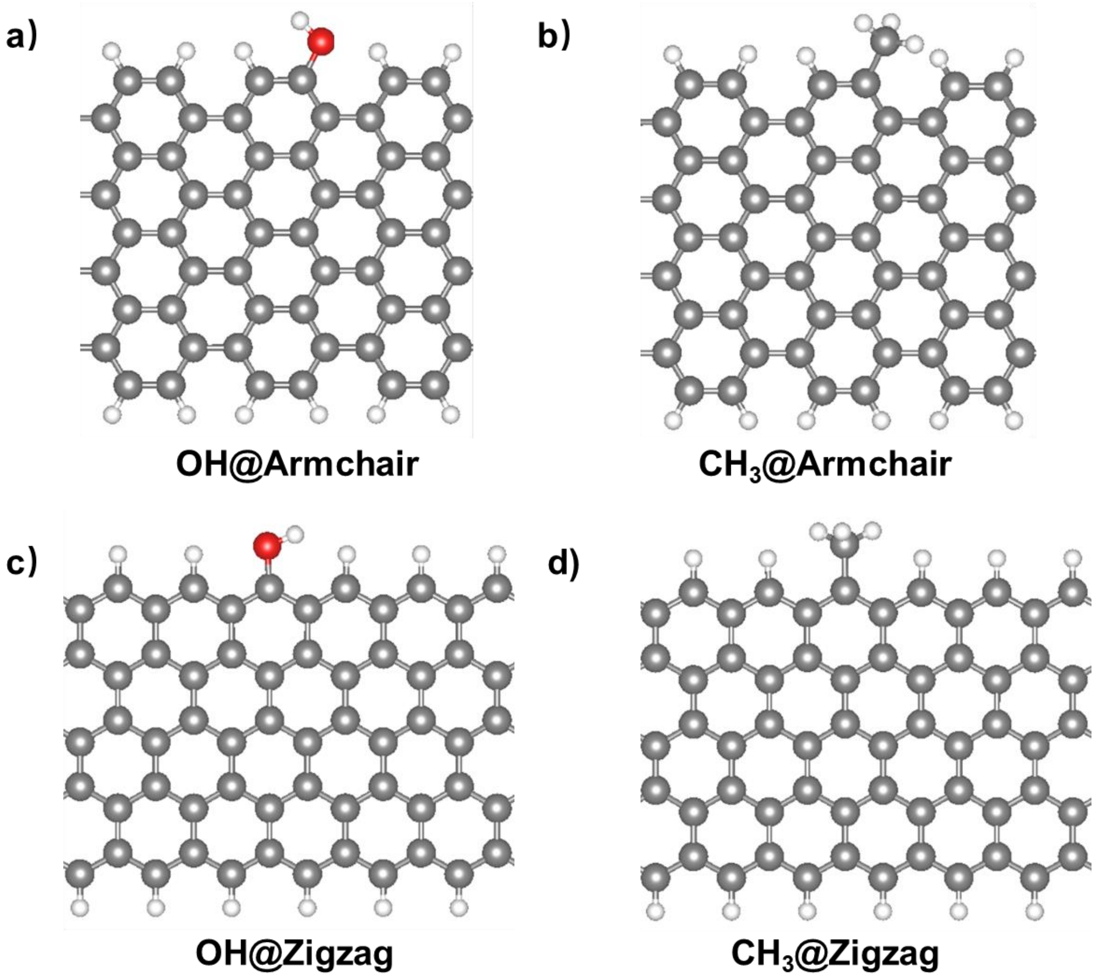

Figure S20. Computational models. (a) OH and (b) CH_3 on graphene ribbons with armchair edges; (c) OH and (d) CH_3 on graphene ribbons with zigzag edges.

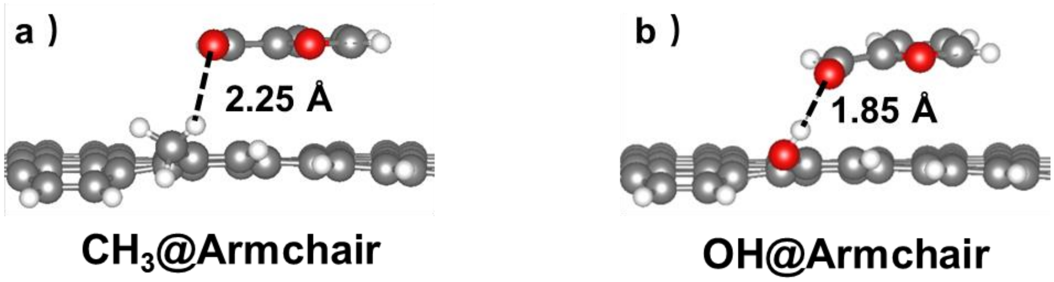

Figure S21. Optimized configurations of furfural adsorption over (a) CH_3 group and (b) OH group on armchair models.

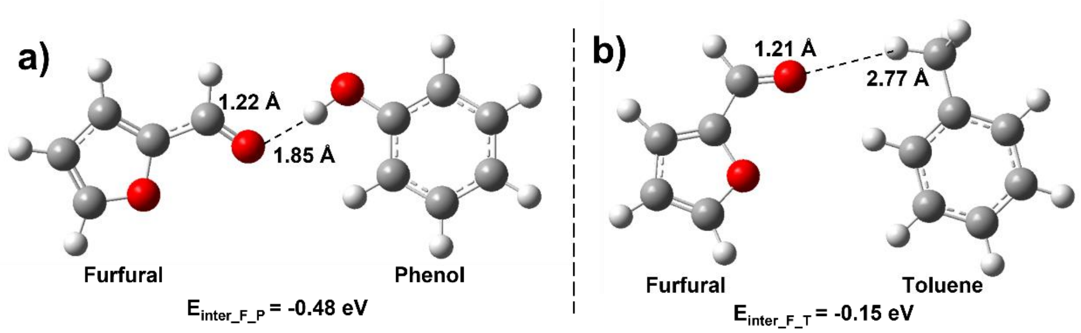

Figure S22. Optimized configurations of the interaction between furfural and monomers. (a) Furfural and Phenol; (b) Furfural and Toluene.

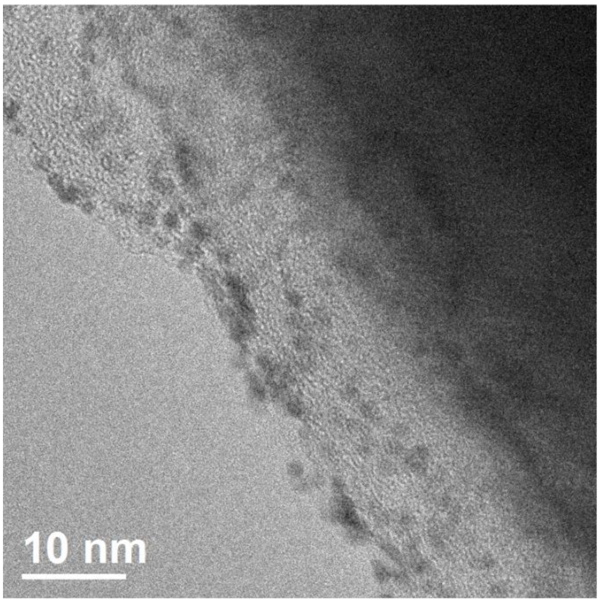

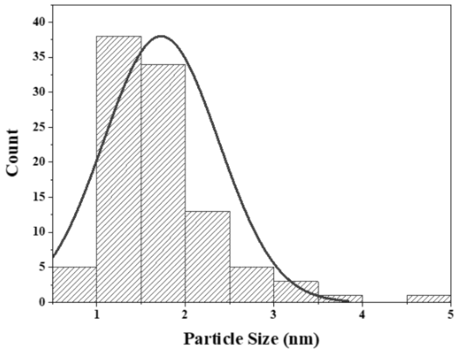

d_{TEM}=2.3 nm
Loading：0.2wt%

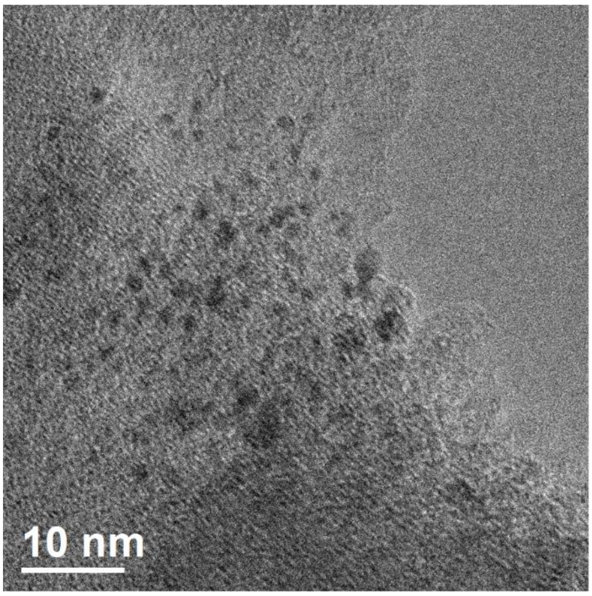

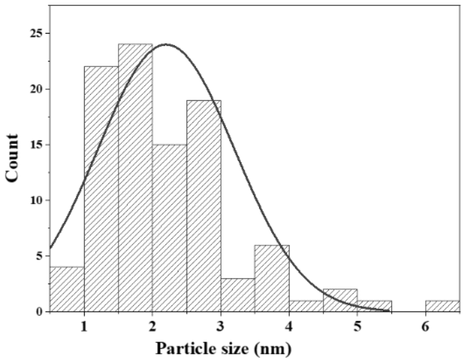

d_{TEM}=3.0 nm
Loading：0.5 wt%

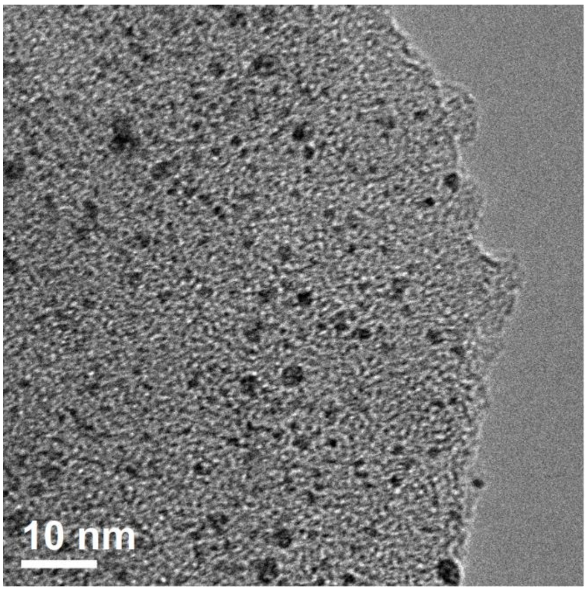

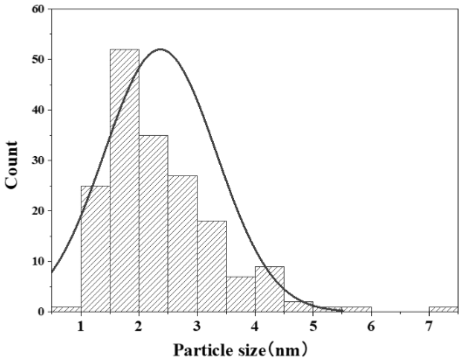

d_{TEM}=3.3 nm
Loading：1 wt%

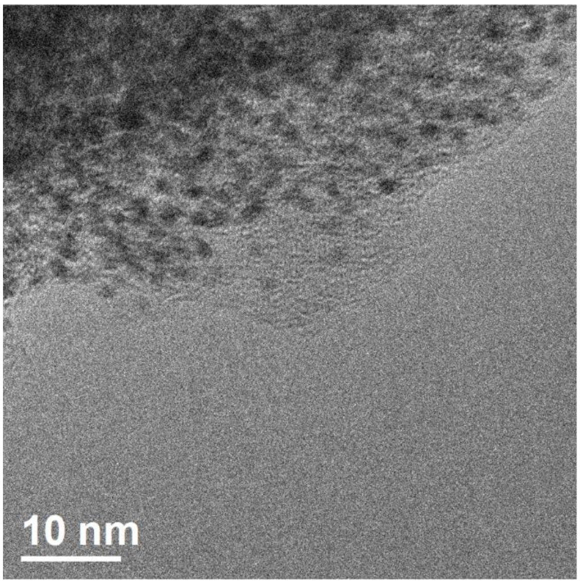

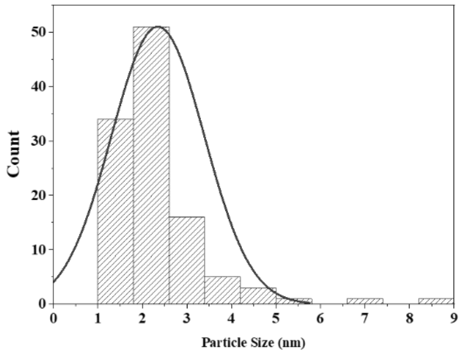

d_{TEM}=3.6 nm
Loading：2 wt%

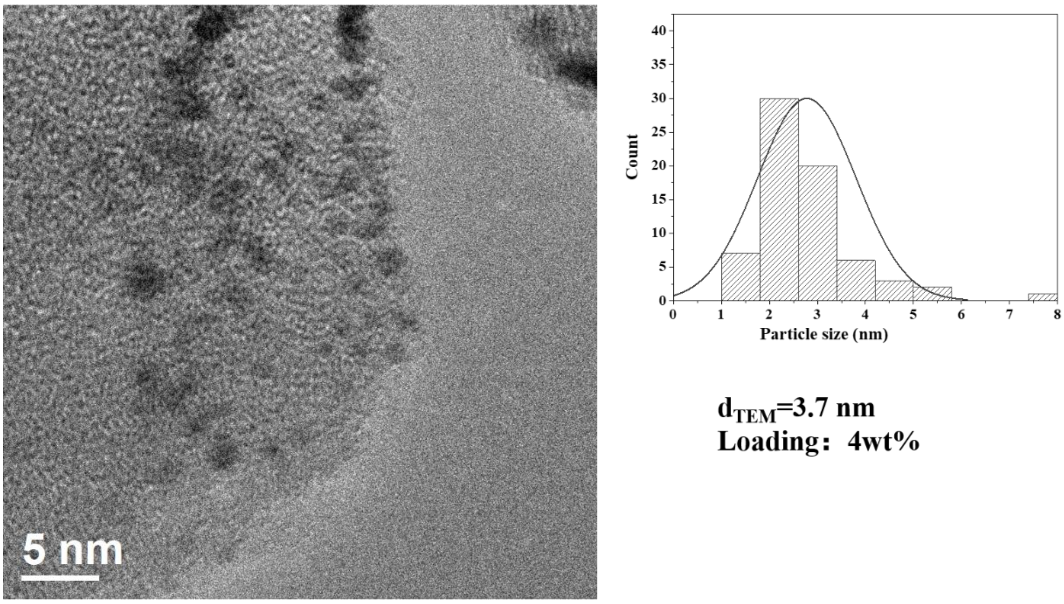

Figure S23. Particle size. TEM and particle size distribution of different Ir loading catalysts.

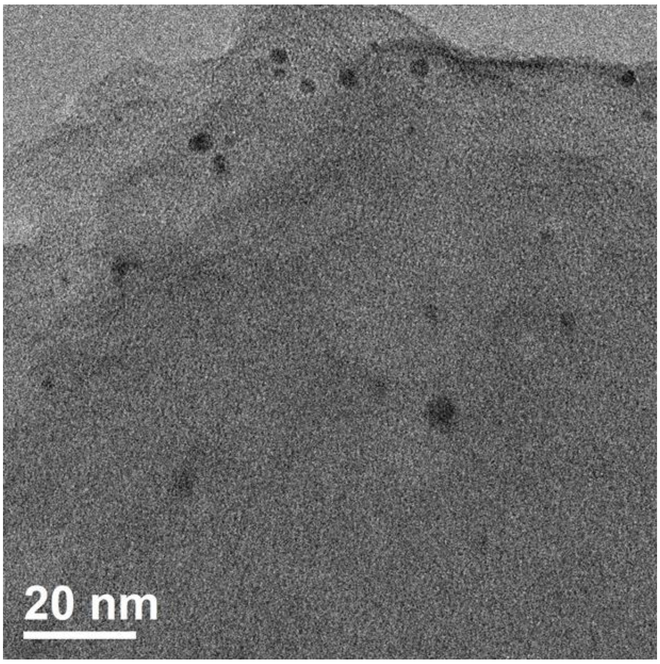

Figure S24. Ir particles after reaction. TEM images of Ir-HCP-OH after reaction.

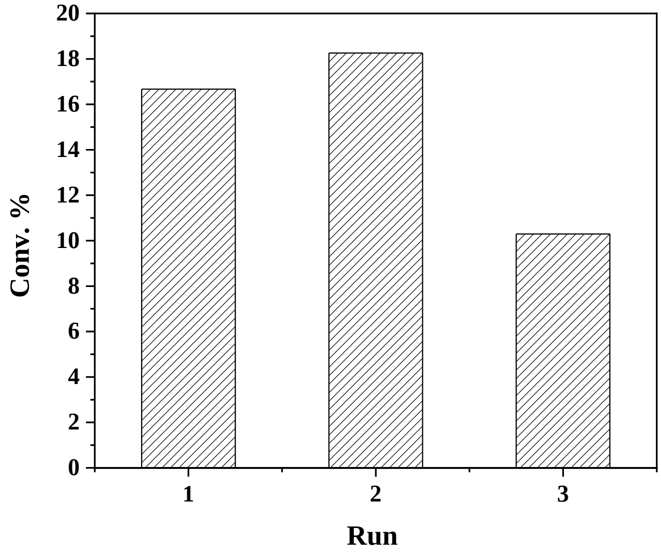

Figure S25. Catalyst reusability of Ir-HCP-OH.

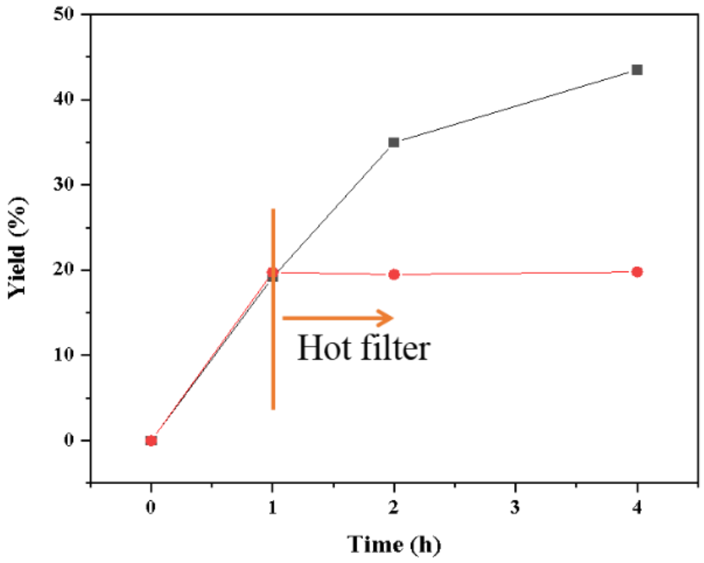

Figure S26. Hot filter experiment of furfural hydrogen with Ir-HCP-OH.

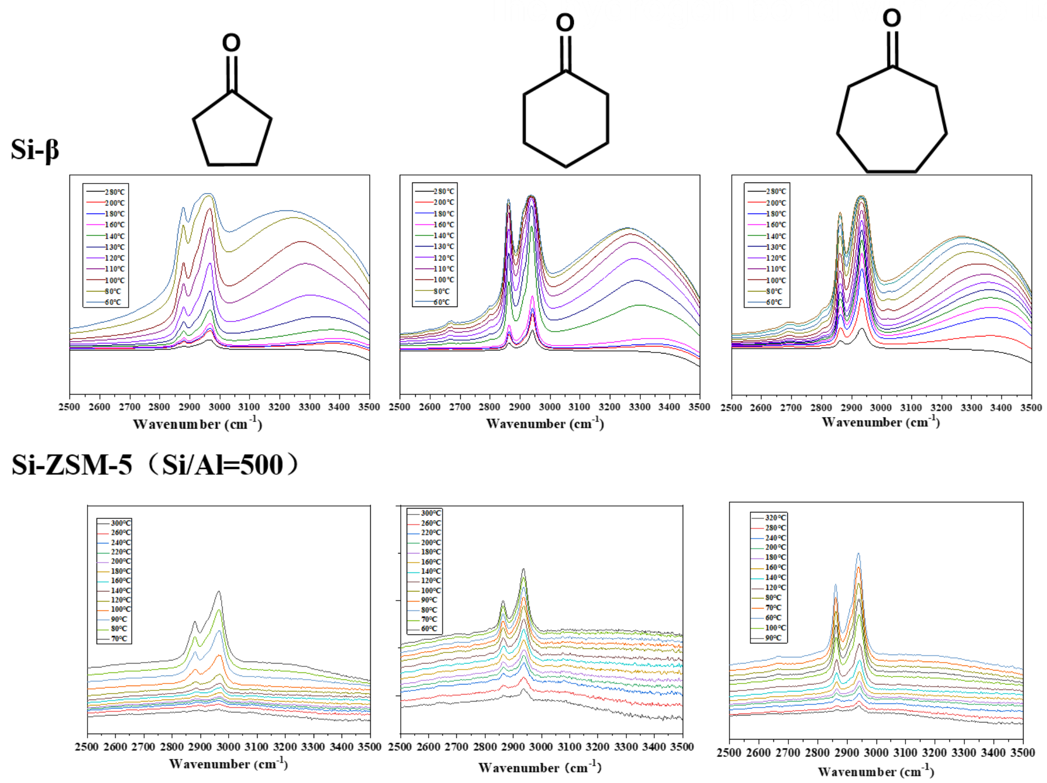

Figure S27. Ketone adsorption difference over different zeolites. *In situ* FT-IR of cyclopentanone, cyclohexanone, and cycloheptanone adsorption on Si-Beta and Si-ZSM-5 and corresponding TPD results.

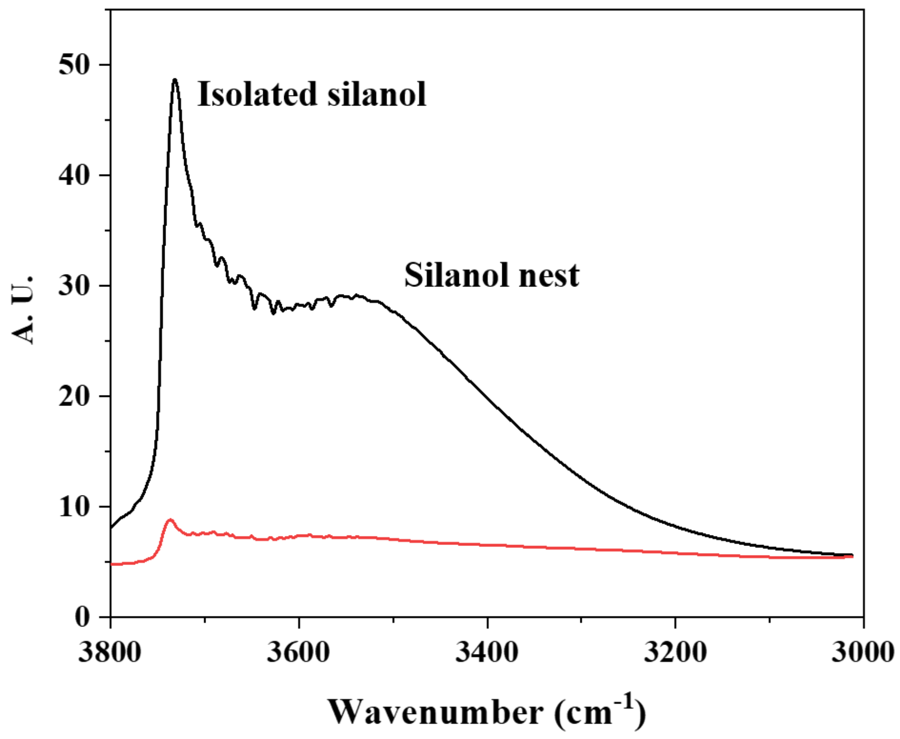

Figure S28. Surface hydroxy difference of Si-Beta and Si-ZSM-5. Drifts-IR after K-M transformation. Peaks at 3780 cm^{-1} assigned to isolated silanol and the broad peaks at 3500 cm^{-1} to the silanol nests.

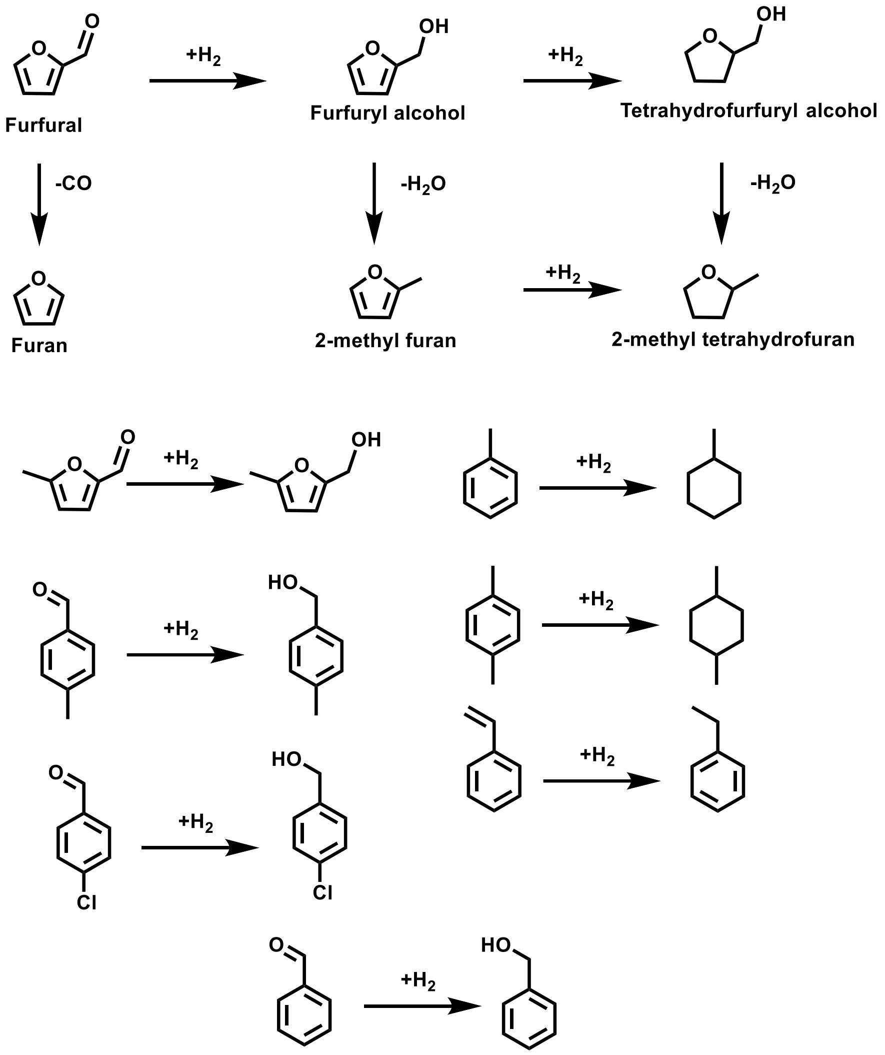

Figure S29. Reaction networks for the reactions depicted in figures 2a, 2c, and 2d.

Table S1. Textural properties of HCP, Ir-HCP, Si-Beta, and ZSM-5.

Sample	S_{BET} (m[2]g[-1]) [a]	D_{pore} (nm) [b]	V_{pore} (cm[3]g[-1])[c]	N (wt%)[d]	C (wt%)[d]	H(wt%)[d]
**HCP-CH_3**	**720**	**2.8**	**0.49**	**0.71**	**76.33**	**6.76**
**HCP-OH**	**324**	**3.5**	**0.29**	**0.70**	**84.54**	**8.64**
**Ir-HCP-CH_3**	**701**	**2.7**	**0.47**	**0.72**	**80.51**	**6.41**
**Ir-HCP-OH**	**253**	**3.8**	**0.24**	**0.77**	**78.90**	**6.78**
**Si-BETA**	**511**	**0.84**	**0.24**	**-**	**-**	**-**
**Si-ZSM-5**	**334**	**0.80**	**0.15**	**-**	**-**	**-**

[a] Calculated with the multipoint BET equation. [b] Pore width calculated with the NLDFT model. [c] Total pore volume calculated at P/P_0 = 0.99. [d] Determined by elemental analysis.

Table S2. Adsorption area of cyclopentanone, cyclohexanone, and cycloheptanone on Si-Beta and Si-ZSM-5.

	**Area (cm[-1])**			**Comparable Area[a]**		
	**Cyclo-pentanone**	**Cyclo-hexanone**	**Cyclo-heptanone**	**Cyclo-pentanone**	**Cyclo-hexanone**	**Cyclo-heptanone**
**Si-Beta**	**74.84**	**84.4**	**89.6**	**92.8**	**84.4**	**166.7**
**Si-ZSM-5**	**9.46**	**7.55**	**2.15**	**11.73**	**7.55**	**3.99**

a, comparable area calculate
cyclopentanone:cyclohexanone:cycloheptanone = 1.23： 1:1.86

Table S3. Adsorption energies (E_{ads}) of furfural over OH group and CH_3 group on the zigzag model and armchair model.

E_{ads} of furfural	Zigzag model	Armchair model
**OH group**	**-0.40 eV**	**-0.47 eV**
**CH_3 group**	**-0.33 eV**	**-0.31 eV**

Table S4. Comparation of recent works on furfural hydrogenation modulation.

Entry	Catalyst	Active site	Main products	Method to modulate catalytic performance	Method	Ref.
1	OH functional HCPs supported Ir	Ir	Furfural alcohol	Hydrogen bond interaction	Experiment + DFT	This work
2	Organic modified Pd/TiO_2	Pd	Furfural alcohol	Surface active site modulation	Experiment	[1]
3	Co-impregnation of Pd and Ir on SiO_2	Ir,Pd	Furfural alcohol and tetrahydrofurfuryl alcohol	Metal alloy method	Experiment	[2]
4	Pt(111), stepped Pt(211), and Pt55 cluster	Pt	Furfural alcohol and furan	Particle size	DFT	[3]
5	sol-immobilization Pd/TiO_2	Pd	Furfural alcohol and tetrahydrofurfuryl alcohol	Particle size and active site	Experiment + DFT	[4]
6	α-MoC	α-MoC	Methyl furan	Solvent	Experiment + DFT	[5]
7	Phosphoric acid modified Pt/Al2O3	Pt	Methyl furan	Interfacial Metal-acid site	Experiment	[6]
8	Pd(111)	Pd	Furfuryl alcohol or furan	Hydrogen-coverage	DFT	[7]

Supplementary Note 1

In order to test the universality of the H bond in other materials except the special designed POPs, we tested the adsorption of ketones on Si- Beta and Si-ZSM-5 using *in-situ* FT-IR (Figure S27). Three different ketones with different molecule size including cyclopentanone, cyclohexanone, and cycloheptanone were tested. Irrespective of the substrate, Si-Beta adsorbs almost 10× than Si-ZSM-5 (Table S2). Although the two zeolites differ in BET surface area, pore volume, and pore size (Table S1), these little differences cannot explain such a huge adsorption difference. DRIFTS-IR reveals a huge difference in the area around 3500 cm^{-1} (Figure S28), ascribed to Si-OH groups, implying many more OH groups in Si-Beta than Si-ZSM-5 that can hydrogen bond with the carbonyl groups. which can form the hydrogen bond between the carbonyl groups and the Si-OH. It is highly believed that this is the main reason for the huge adsorption difference. This experiment demonstrated weak interaction like H bond might widely existed in during the

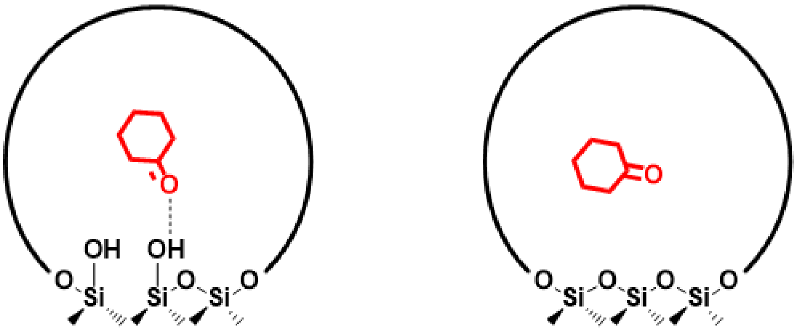

Illustration of hydrogen bond formed between ketone and Si-OH in zeolite pores.

Supplementary References

1. Pang SH, Schoenbaum CA, Schwartz DK, Medlin JW. Effects of Thiol Modifiers on the Kinetics of Furfural Hydrogenation over Pd Catalysts. ACS Catalysis 4, 3123-3131 (2014).

2. Nakagawa Y, Takada K, Tamura M, Tomishige K. Total Hydrogenation of Furfural and 5-Hydroxymethylfurfural over Supported Pd–Ir Alloy Catalyst. ACS Catalysis 4, 2718-2726 (2014).

3. Cai Q-X, Wang J-G, Wang Y-G, Mei D. Mechanistic insights into the structure-dependent selectivity of catalytic furfural conversion on platinum catalysts. AIChE Journal 61, 3812-3824 (2015).

4. Rogers SM, et al. Tandem Site- and Size-Controlled Pd Nanoparticles for the Directed Hydrogenation of Furfural. ACS Catalysis 7, 2266-2274 (2017).

5. Deng Y, et al. Solvent Tunes the Selectivity of Hydrogenation Reaction over α-MoC Catalyst. Journal of the American Chemical Society 140, 14481-14489 (2018).

6. Zhang J, et al. Control of interfacial acid–metal catalysis with organic monolayers. Nature Catalysis 1, 148-155 (2018).

7. Wang S, Vorotnikov V, Vlachos DG. Coverage-Induced Conformational Effects on Activity and Selectivity: Hydrogenation and Decarbonylation of Furfural on Pd(111). ACS Catalysis 5, 104-112 (2015).
